# SGLT2 inhibitors and outcomes in the chronic Post-LVAD phase: a multicenter real-world cohort study

**DOI:** 10.3389/fphar.2026.1922067

**Published:** 2026-09-08

**Authors:** Jheng-Yan Wu, Keng-Wei Lee, Sheng-Chi Huang, Hsuan-Yuan Chang, Yu-Min Lin

**Affiliations:** 1 Department of Nutrition, Chi Mei Medical Center, Tainan, Taiwan; 2 Department of Public Health, College of Medicine, National Cheng Kung University, Tainan, Taiwan; 3 Division of Cardiology, Department of Internal Medicine, Chi Mei Hospital, Chiali, Tainan, Taiwan; 4 Department of Medical Education, Chi Mei Medical Center, Tainan, Taiwan; 5 Division of Hepatogastroenterology, Department of Internal Medicine, Chi Mei Medical Centre, Tainan, Taiwan; 6 Division of Cardiology, Department of Internal Medicine, Chi Mei Medical Center, Tainan, Taiwan

**Keywords:** all-cause mortality, heart failure, heart failure exacerbation, left ventricular assist device, major adverse cardiovascular events, real-world evidence

## Abstract

**Background:**

The effect of sodium–glucose cotransporter-2 inhibitors (SGLT2i) in patients with left ventricular assist devices (LVAD) remains uncertain. We evaluated the association between SGLT2i use and adverse outcomes during the chronic post-LVAD phase.

**Methods:**

We conducted a retrospective cohort study using the TriNetX network. Adults with LVAD implantation (2014–2025) who survived ≥90 days were included. SGLT2i exposure (empagliflozin or dapagliflozin) was identified by prescription records. A prespecified 90-day delayed outcome window defined follow-up from 3 months to 1 year after the index date. The primary outcome was a composite of all-cause mortality or heart failure exacerbation (HFE). Secondary outcomes included HFE, all-cause mortality, and major adverse kidney events (MAKE). Propensity score matching (1:1) and Cox regression were applied.

**Results:**

After matching, 1,318 patients were included in each group. SGLT2i use was associated with a lower risk of the primary composite endpoint (20.0% vs. 30.6%; HR 0.65; 95% CI 0.56–0.76; p < 0.001). Risks of HFE (18.4% vs. 26.9%; HR 0.69; p < 0.001), all-cause mortality (4.2% vs. 7.3%; HR 0.60; p = 0.002), and MAKE (27.5% vs. 35.1%; HR 0.81; p = 0.003) were also lower. Safety outcomes were similar, including urinary tract infection (HR 0.69; p = 0.091) and diabetic ketoacidosis (HR 1.25; p = 0.437).

**Conclusion:**

SGLT2i use was associated with lower risks of cardiovascular and kidney events in patients during the chronic post-LVAD phase, without increased adverse events. These findings support further evaluation of SGLT2i as part of long-term management after LVAD.

## Introduction

Heart failure (HF) remains a major and growing global health burden, affecting more than 64 million individuals worldwide. In the United States, approximately 6.7 million adults are currently living with HF, and this number is projected to increase substantially over the coming decades (Bozkurt et al., 2025; Savarese et al., 2023). Despite substantial advances in pharmacologic and device-based therapies, a subset of patients progresses to advanced HF and may ultimately require heart transplantation or durable mechanical circulatory support. Contemporary data from the Society of Thoracic Surgeons Intermacs registry included 29,634 continuous-flow left ventricular assist device (LVAD) implantations between 2014 and 2024, reflecting the expanding contemporary experience with durable LVAD support (Meyer et al., 2025). Despite advances in pharmacologic and device-based therapies, a large proportion of patients continue to progress to advanced HF with worsening left ventricular systolic function despite maximal guideline-directed medical therapy. The left ventricular assist device (LVAD) has emerged as a pivotal treatment option for such patients, either as a bridge to transplantation or as destination therapy (Jefferson et al., 2021).

Landmark trials comparing LVAD implantation with optimal medical therapy in patients with New York Heart Association (NYHA) class IV HF demonstrated a 48% reduction in all-cause mortality (Rose et al., 2001). Nevertheless, LVAD-associated morbidity and mortality remains substantial, with recurrent heart failure exacerbation (HFE) and acute kidney injury representing the most frequent adverse events (Shah et al., 2022; Mehra et al., 2021; Pretorius et al., 2025).

Previous observational studies have demonstrated that continued use of neurohormonal blockade after LVAD implantation, such as β-blockers, renin–angiotensin system inhibitors, or mineralocorticoid receptor antagonists, is associated with lower mortality and improved survival and quality of life (McCullough et al., 2020). In contrast, the role of newer heart failure therapies, particularly sodium–glucose cotransporter 2 inhibitors (SGLT2is), has been rarely explored in this population. SGLT2is have been shown to confer significant prognostic benefits across a broad spectrum of conditions, including heart failure, metabolic disorders, and chronic kidney disease, through mechanisms involving hemodynamic, metabolic, and anti-inflammatory effects (Wang et al., 2025; Kumar et al., 2025). An observational study have suggested potential benefits of SGLT2is in patients with LVAD, yet these analyses were limited by early study periods, small sample sizes, and restricted generalizability (Pham et al., 2025).

To address this knowledge gap, we conducted a study using the TriNetX Research Network, a large multi-institutional electronic health record platform to examine the association between SGLT2i use and clinical outcomes in patients who underwent LVAD implantation and reached a stable post-implantation phase. The primary outcome was a composite of all-cause mortality or HFE between 3 months and 1 year after LVAD implantation, while secondary outcomes included HFE, all-cause mortality, and major adverse kidney events (MAKE). The findings of this study aim to provide real-world evidence to inform post-LVAD pharmacologic strategies and to clarify the potential therapeutic role of SGLT2is in this high-risk population.

## Methods

### Data source

This retrospective cohort study was conducted using data from the TriNetX platform, a global federated health research network that integrates electronic health records from approximately 187 million patients across 162 healthcare organizations (HCOs). The database provides detailed information on diagnoses, procedures, medications, laboratory findings, and genomic data. The present study used only de-identified, aggregate-level data available through the TriNetX research network, with no direct patient contact or study-specific intervention. The study was classified as exempt from institutional review board review by the Western Institutional Review Board. The study design and reporting adhered to the Strengthening the Reporting of Observational Studies in Epidemiology (STROBE) guidelines.

### Study design

We included adult patients aged 18 years or older who underwent LVAD implantation between 1 January 2014 and 30 September 2025. LVAD implantation was identified using the ICD-10-PCS codes 02HA3QZ, 02HA0QZ, and 02HA4QZ, together with the CPT codes 33975 and 33979. Patients were classified into two groups. The SGLT2i group comprised individuals who had received SGLT2is, identified through the RxNorm codes 1545653 for empagliflozin and 1488564 for dapagliflozin. The control group included patients who did not receive SGLT2i therapy. For those in the SGLT2i group, the index date was defined as the date of the SGLT2i prescription, while for those in the control group, it was defined as the date of LVAD implantation. The baseline period was the 12 months preceding the index date. Detailed definitions and coding algorithms for patient demographics, clinical characteristics, diagnoses, procedures, medications, and laboratory data are summarized in Supplementary Table S2.

### Covariates and propensity score matching

After defining the study cohorts, index dates, outcomes, and potential confounders, we constructed a covariate matrix based on patient-level data from the 1-year period before the index date. Propensity scores were calculated using logistic regression to estimate the likelihood of each patient receiving SGLT2i according to baseline characteristics. A 1:1 greedy nearest-neighbor matching approach without replacement was performed, with the caliper width set at 0.1 times the pooled standard deviation of the logit-transformed propensity score. Covariate balance between the two groups was considered acceptable when the standardized mean difference (SMD) for all variables was less than 0.1 (Haukoos and Lewis, 2015).

The propensity score matching (PSM) model incorporated a comprehensive set of baseline covariates to ensure balanced comparison between the study groups. Demographic variables included age (years, mean [SD]), sex (female and male), and race (White, Black or African American, and unknown). Clinical comorbidities comprised atrial fibrillation and flutter, chronic kidney disease, type 2 diabetes mellitus, type 2 diabetes mellitus with kidney complications, nicotine dependence, chronic obstructive pulmonary disease, type 2 diabetes mellitus with neurological complications, type 2 diabetes mellitus with circulatory complications, cirrhosis of the liver, and type 2 diabetes mellitus with ophthalmic complications. Baseline medication use was also assessed, including platelet aggregation inhibitors, beta-blockers, aldosterone antagonists and other potassium-sparing agents, angiotensin II inhibitors (ARBs), angiotensin-converting enzyme inhibitors (ACEis), Sacubitril/Valsartan, and glucagon-like peptide-1 analogues. Laboratory and clinical parameters consisted of systolic blood pressure (mmHg, mean [SD] and proportion <160), low-density lipoprotein cholesterol (LDL-C, mg/dL, mean [SD] and proportion <130), estimated glomerular filtration rate (eGFR, mL/min/1.73 m^2^, mean [SD] and proportion <15), and hemoglobin A1c (HbA1c, %, mean [SD] and proportion <9). SBP was used as the available structured blood pressure variable for covariate adjustment. Mean arterial pressure and information regarding the method of blood pressure measurement (e.g., Doppler-based, oscillometric, or invasive measurements) were not consistently available across participating healthcare organizations. No investigator-level imputation of missing laboratory or vital-sign measurements was performed. Because the aggregate TriNetX analytical environment did not provide patient-level missingness denominators within the matched cohorts, variable-specific missingness could not be quantified by treatment group.

The complete list of covariates entered into the propensity score model, together with their corresponding codes and functional forms, is provided in Supplementary Table S2.

### Outcomes and follow-up

The primary outcome of this study was a composite endpoint of all-cause mortality or HFE. Secondary outcomes included the individual components of the primary composite endpoint (all-cause mortality and HFE) and MAKE. HFE was operationally defined as an event requiring administration of injectable diuretics or presentation with acute pulmonary edema. MAKE was operationally defined as the occurrence of hemodialysis, end-stage kidney disease, or CKD stage 5, or acute kidney injury. To assess the robustness of the findings, hernia was selected as a negative control outcome because no established biological association with SGLT2i therapy is currently recognized, making it useful for assessing potential residual confounding. In addition, we evaluated safety outcomes, including urinary tract infection (UTI) and diabetic ketoacidosis (DKA). The follow-up period for all outcomes began 90 days after the index date and continued until the first occurrence of an outcome event, the last recorded clinical encounter, death, or 1 year after the index date, whichever occurred first. Outcomes were assessed within a prespecified post-index window beginning 90 days after the index date and continuing through 1 year. This 90-day delayed outcome window was selected to reduce the influence of perioperative complications and early postoperative hemodynamic instability. The coding algorithms used to identify these outcomes are provided in Supplementary Table S3.

### Subgroup analysis

For the primary outcome, subgroup analyses were conducted in the overall study population and further stratified by sex (female or male) and age category (18–64 years or 65 years and older). Additional subgroup analyses were performed according to the presence or absence of chronic kidney disease, type 2 diabetes, and coronary artery disease. The analyses also examined baseline use of ACEIs or ARBs, beta-blockers, and mineralocorticoid receptor antagonists (MRAs). For each subgroup analysis, the subgroup population was defined independently from the source cohort, followed by separate 1:1 propensity score matching using the same prespecified covariates as in the primary analysis. Therefore, the subgroup-specific matched cohorts were distinct analytic populations and were not subsets obtained by partitioning the primary propensity-matched cohort. Subgroup analyses were considered exploratory, and formal interaction tests were not performed.

### Statistical analysis

Continuous variables were expressed as means with standard deviations, and categorical variables were presented as counts with corresponding percentages. PSM was performed for the primary analysis and repeated independently within each prespecified subgroup analysis. Cox proportional hazards regression models were used to estimate hazard ratios (HRs) and 95% confidence intervals (CIs). The proportional hazards assumption was assessed using Schoenfeld residuals and was not violated (P > 0.05). Time-to-event outcomes were analyzed using Kaplan–Meier survival curves, and group differences were evaluated with the log-rank test. To examine the potential influence of unmeasured confounders, E-values were calculated (VanderWeele and Ding, 2017). Additionally, an era-restricted sensitivity analysis was conducted by stratifying the cohort into two periods (2014–2019 and 2020–2025) to account for temporal changes in LVAD management, including evolving device technology and antithrombotic strategies. All statistical analyses were conducted using the TriNetX analytical platform.

### Sensitivity analysis

To assess the potential influence of delayed treatment initiation and temporal misalignment between LVAD implantation and SGLT2i initiation, we restricted the SGLT2i group to patients who initiated treatment within 1 month and within 2 months after LVAD implantation. The individual components of MAKE were analyzed separately to assess whether the composite kidney outcome was driven by a specific component.

## Results

### Study cohort

**FIGURE 1:**
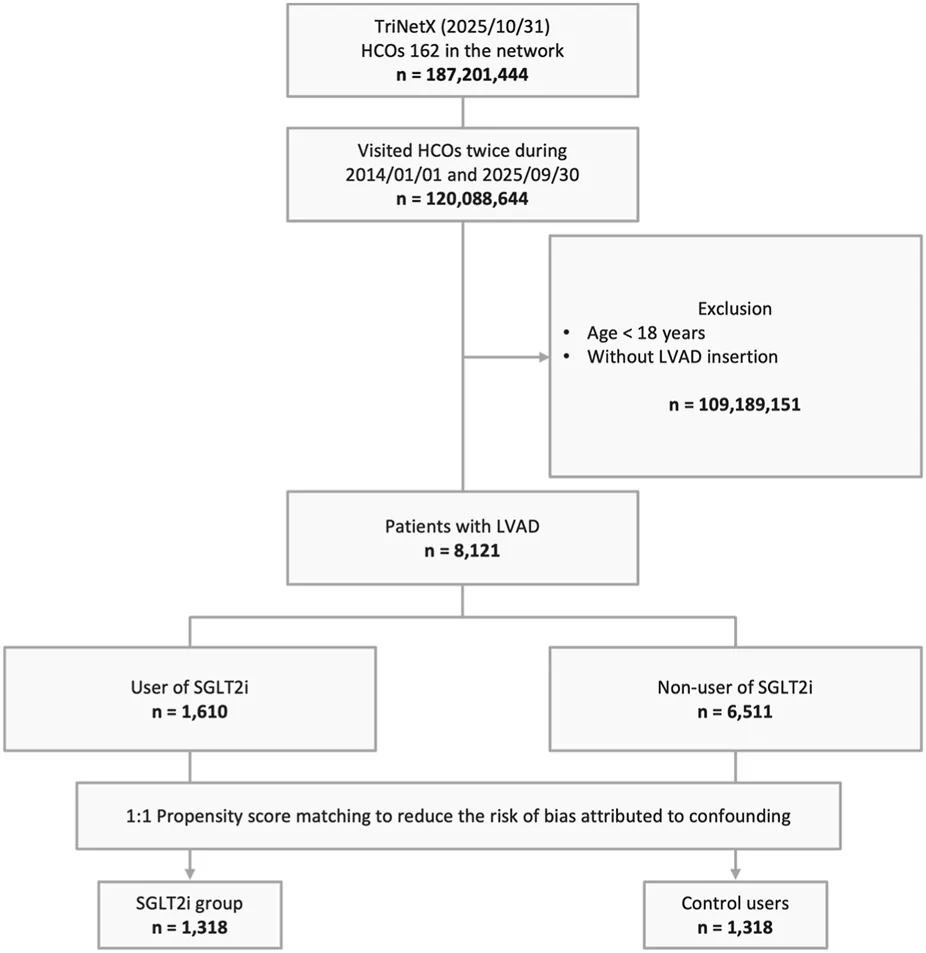
Study cohort process. HCOs, healthcare organizations; LVAD, left ventricular assist device; SGLT2i, sodium-glucose cotransporter 2 inhibitor.

A total of 8,121 patients with LVAD implantation were identified during the study period. Of these, 1,610 received SGLT2i therapy and 6,511 did not. After 1:1 PS M, two well-balanced cohorts comprising 1,318 patients each were included in the final analysis (Figure 1).

### Characteristics of study subjects

Prior to matching, patients who received SGLT2i therapy and those who did not were generally similar in age (mean 55.7 ± 14.0 vs. 55.4 ± 15.2 years) and sex distribution (male: 77.3% vs. 76.3%; Table 1). The racial composition showed a slightly higher proportion of White patients in the control group, whereas the SGLT2i group included a greater proportion of Black or African American patients and fewer individuals with unknown race. Before matching, comorbid conditions such as atrial fibrillation and flutter, chronic kidney disease, and type 2 diabetes mellitus were more frequently documented among patients in the SGLT2i group (Table 1). After matching, the distributions of these comorbidities achieved acceptable balance. Medication use patterns before matching indicated higher frequencies of platelet aggregation inhibitors, beta-blockers, aldosterone antagonists, angiotensin II inhibitors, and glucagon-like peptide-1 analogues in the SGLT2i group. Following matching, the proportions of patients using these medications were comparable between groups. Baseline laboratory and clinical measurements, including systolic blood pressure, LDL-C, eGFR, and HbA1c, were also well balanced after matching (Table 1). The mean systolic blood pressure was 93.8 ± 18.6 mmHg in the SGLT2i group and 98.0 ± 22.9 mmHg in the control group. The mean eGFR values were 74.2 ± 34.4 and 58.9 ± 24.9 mL/min/1.73 m^2^, respectively, while the mean HbA1c levels were 6.5% ± 1.4% and 6.6% ± 1.4%. After propensity score matching, most categorical baseline characteristics achieved acceptable balance; however, residual imbalance remained in several continuous variables, particularly eGFR (74.2 ± 34.4 vs. 58.9 ± 24.9 mL/min/1.73 m^2^; SMD 0.509), as well as systolic blood pressure (SMD 0.201) and HbA1c (SMD 0.133). In contrast, the prevalence of severe kidney dysfunction defined as eGFR <15 mL/min/1.73 m^2^ was similar between groups (9.4% vs. 9.6%; SMD 0.008) (Table 1).

**TABLE 1:** Baseline characteristics of the SGLT2i and the control groups before and after matching.

Variables	Before matching			After matching		
	SGLT2i group (n = 1,610)	Control group (n = 6,511)	Standardized difference	SGLT2i group (n = 1,318)	Control group (n = 1,318)	Standardized difference
Age, years						
Mean (SD)	55.7 (14)	55.4 (15.2)	0.019	55.7 (14.3)	56 (13.6)	0.017
Sex, n (%)						
Female	366 (22.7)	1,531 (23.7)	0.022	300 (22.8)	298 (22.6)	0.004
Male	1,244 (77.3)	4,934 (76.3)	0.022	1,018 (77.2)	1,020 (77.4)	0.004
Race, n (%)						
White	904 (56.1)	3,870 (59.9)	0.075	764 (58)	764 (58)	0
Black or african american	487 (30.2)	1,463 (22.6)	0.173	372 (28.2)	376 (28.5)	0.007
Unknown	90 (5.6)	795 (12.3)	0.237	83 (6.3)	77 (5.8)	0.019
Comorbidities, n (%)						
Atrial fibrillation and flutter	1,038 (64.5)	3,300 (51)	0.274	831 (63.1)	828 (62.8)	0.005
Chronic kidney disease	1,023 (63.5)	3,081 (47.7)	0.324	796 (60.4)	802 (60.9)	0.009
Type 2 diabetes mellitus	1,018 (63.2)	2,362 (36.5)	0.554	750 (56.9)	747 (56.7)	0.005
Type 2 diabetes mellitus with kidney complications	637 (39.6)	1,011 (15.6)	0.556	426 (32.3)	421 (31.9)	0.008
Nicotine dependence	402 (25)	949 (14.7)	0.26	292 (22.2)	270 (20.5)	0.041
Chronic obstructive pulmonary disease	322 (20)	889 (13.8)	0.167	239 (18.1)	238 (18.1)	0.002
Type 2 diabetes mellitus with neurological complications	280 (17.4)	509 (7.9)	0.289	188 (14.3)	181 (13.7)	0.015
Type 2 diabetes mellitus with circulatory complications	243 (15.1)	317 (4.9)	0.345	152 (11.5)	143 (10.9)	0.022
Cirrhosis of liver	83 (5.2)	207 (3.2)	0.098	66 (5)	65 (4.9)	0.003
Type 2 diabetes mellitus with ophthalmic complications	90 (5.6)	170 (2.6)	0.15	65 (4.9)	58 (4.4)	0.025
Medications, n (%)						
Platelet aggregation inhibitors	1,430 (88.8)	3,959 (61.2)	0.672	1,145 (86.9)	1,160 (88)	0.034
Beta blockers	1,397 (86.8)	4,374 (67.7)	0.468	1,127 (85.5)	1,150 (87.3)	0.051
Aldosterone antagonists and other potassium-sparing agents	1,377 (85.5)	3,415 (52.8)	0.757	1,091 (82.8)	1,077 (81.7)	0.028
Angiotensin II inhibitors	1,260 (78.3)	1,872 (29)	1.137	973 (73.8)	983 (74.6)	0.017
ACEis	430 (26.7)	2,548 (39.4)	0.273	406 (30.8)	404 (30.7)	0.003
Sacubitril/Valsartan	843 (52.4)	866 (13.3)	0.917	570 (43.2)	569 (43.4)	0.006
Glucagon-like peptide-1 analogues	168 (10.4)	100 (1.5)	0.381	75 (5.7)	71 (5.4)	0.013
Systolic blood pressure, mmHg						
Mean (SD)	94.3 (19)	98.1 (24.1)	0.177	93.8 (18.6)	98 (22.9)	0.201
<160 mmHg	1,406 (87.3)	4,545 (70.3)	0.426	1,136 (86.2)	1,159 (87.9)	0.052
Cholesterol in LDL, mg/dL						
Mean (SD)	69.8 (33.7)	73 (34.8)	0.092	70.5 (34.2)	72.7 (35.7)	0.062
<130 mg/dL	1,194 (74.2)	3,821 (59.1)	0.324	969 (73.5)	981 (74.4)	0.021
eGFR, mL/min/1.73 m^2^						
Mean (SD)	73.2 (34.2)	60.9 (28.4)	0.39	74.2 (34.4)	58.9 (24.9)	0.509
<15 mL/min/1.73 m^2^	153 (9.5)	681 (10.5)	0.034	124 (9.4)	127 (9.6)	0.008
HbA1c, %						
Mean (SD)	6.6 (1.5)	6.4 (1.5)	0.118	6.5 (1.4)	6.6 (1.4)	0.133
<9%	1,223 (76)	4,120 (63.7)	0.269	998 (75.7)	1,005 (76.3)	0.012

ACEis, angiotensin-converting enzyme inhibitors; ARNI, angiotensin receptor-neprilysin inhibitor; eGFR, estimated Glomerular filtration rate; HbA1c, hemoglobin A1c; LDL, low-density lipoprotein; SD, standard deviation.

### Primary and secondary outcomes

**FIGURE 2:**
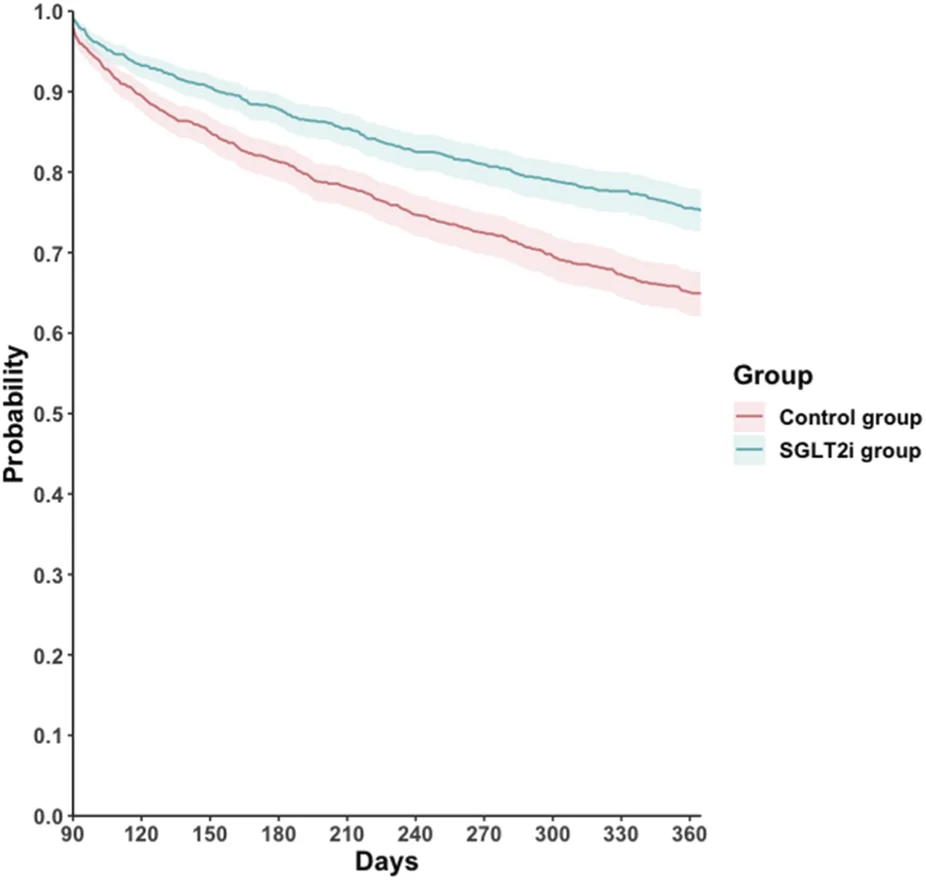
Kaplan–Meier curves for freedom from the primary composite endpoint in the SGLT2i and control groups. The x-axis represents days from the index date, defined as the date of SGLT2i prescription for the SGLT2i group and the date of LVAD implantation for the control group. The curves were generated directly by the TriNetX analytical platform using the original index-date time scale; therefore, the 90-day landmark was not re-indexed to day 0 on the displayed x-axis. SGLT2i, sodium-glucose cotransporter 2 inhibitor.

Table 2 summarizes the incidence rates and hazard ratios for the primary and secondary outcomes between the SGLT2i and control groups after PSM. During the follow-up period, the incidence of the primary composite endpoint was 20.0% in the SGLT2i group and 30.6% in the control group, with an HR of 0.65–0.76; p < 0.001). For secondary outcomes, HFE occurred in 18.4% of patients in the SGLT2i group and 26.9% of those in the control group (HR, 0.69; 95% CI, 0.58–0.81; p < 0.001). All-cause mortality was observed in 4.2% and 7.3% of patients in the SGLT2i and control groups, respectively (HR, 0.60; 95% CI, 0.43–0.83; p = 0.002). MAKE were reported in 27.5% of the SGLT2i group and 35.1% of the control group (HR, 0.81; 95% CI, 0.71–0.93; p = 0.003). The corresponding E-values for these outcomes ranged from 1.8 to 2.7, indicating robustness to potential unmeasured confounding. For primary outcome, Kaplan-Meier curves consistently demonstrated a higher event-free probability in the SGLT2i group than the control group (log-rank p < 0.001, Figure 2).

**TABLE 2:** Hazard ratio of outcomes between the SGLT2i and the control groups.

Outcome	SGLT2i group (n = 1,318)	Control group (n = 1,318)	HR (95% CI)	*P* value	E-value (95% LCL)
	Events (%)	Events (%)			
Primary outcome					
Composite of all-cause mortality or HFE	264 (20.0)	403 (30.6)	0.65 (0.56,0.76)	<0.001	2.5 (2.0)
Secondary outcomes					
HFE	243 (18.4)	355 (26.9)	0.69 (0.58,0.81)	<0.001	2.3 (1.8)
All-cause mortality	55 (4.2)	96 (7.3)	0.60 (0.43,0.83)	0.002	2.7 (1.7)
MAKE	372 (27.5)	463 (35.1)	0.81 (0.71,0.93)	0.003	1.8 (1.4)

CI, confidence interval; HFE, heart failure exacerbation; HR, hazard ratio; LCL, lower confidence limit; MAKE, major adverse kidney event; SGLT2i, sodium-glucose cotransporter 2 inhibitor.

As shown in Supplementary Table S4, safety and negative control analyses revealed no statistically significant differences between groups. The HR for UTI was 0.69 (95% CI, 0.45–1.06; p = 0.091), and for DKA was 1.25 (95% CI, 0.71–2.23; p = 0.437). The risk of hernia showed an HR of 0.96 (95% CI, 0.73–1.26; p = 0.748), indicating no apparent residual confounding. In era-restricted sensitivity analyses, the association between SGLT2i use and reduced risk of the primary outcome remained consistent across time periods (2014–2019: HR 0.60; 95% CI 0.50–0.72; 2020–2025: HR 0.62; 95% CI 0.54–0.72; both p < 0.001) (Supplementary Table S5).

### Subgroup analysis

**FIGURE 3:**
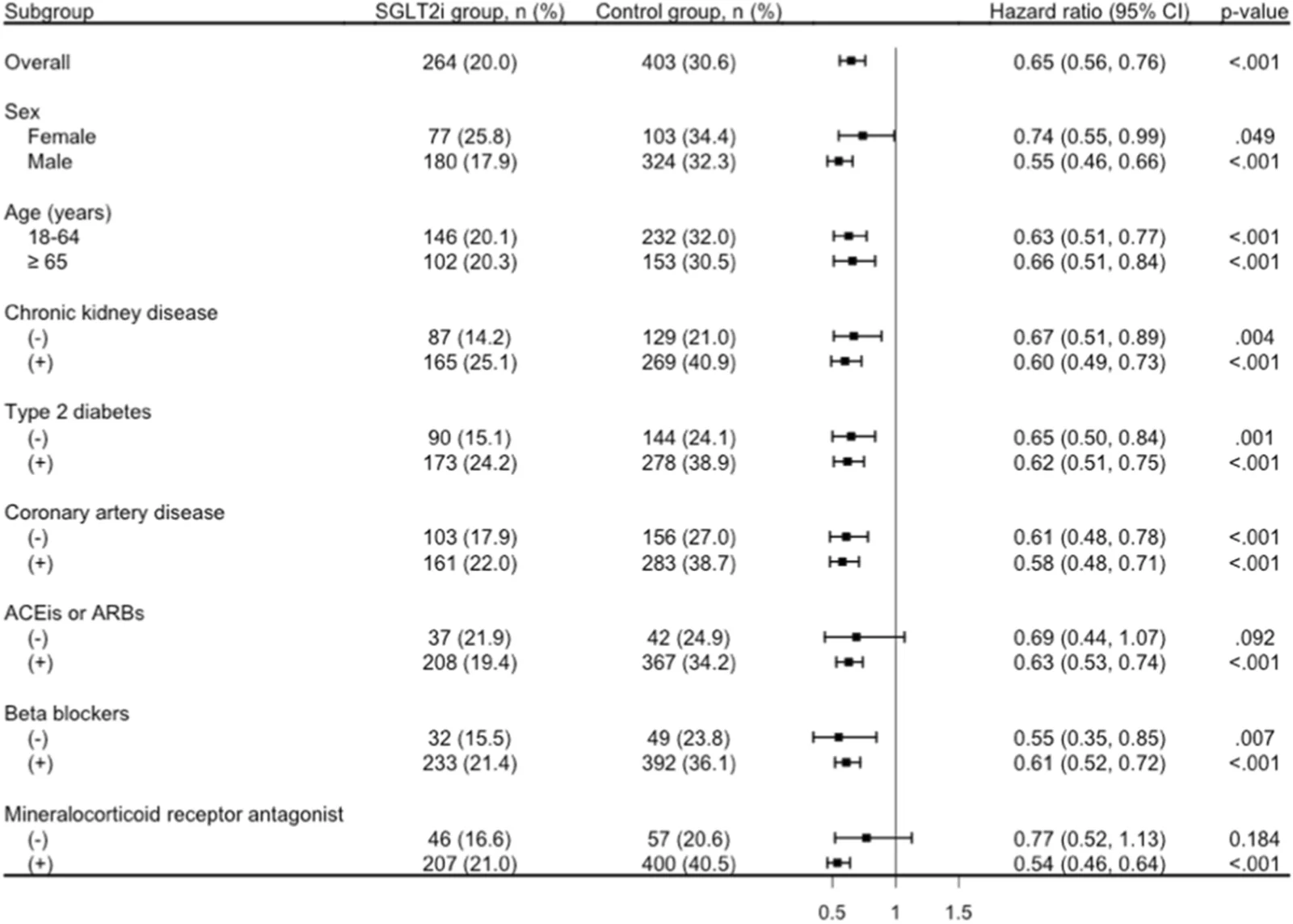
Subgroup-specific propensity-matched analyses of the primary composite endpoint comparing SGLT2i and control groups. Propensity score matching was repeated independently within each subgroup; therefore, subgroup event counts do not represent a partition of the primary matched cohort. Type 2 diabetes was defined using ICD-10-CM code E11. ACEis, angiotensin-converting enzyme inhibitors; ARBs, angiotensin receptor blockers; CI, confidence interval; SGLT2i, sodium-glucose cotransporter 2 inhibitor.

Figure 3 presents the results of subgroup analyses examining the association between SGLT2i use and the primary outcome. Among females, the HR was 0.74 (95% CI, 0.55–0.99; p = 0.049), and among males, the HR was 0.55 (95% CI, 0.46–0.66; p < 0.001). In analyses stratified by age, the HRs were 0.63 (95% CI, 0.51–0.77; p < 0.001) for participants aged 18–64 years and 0.66 (95% CI, 0.51–0.84; p < 0.001) for those aged 65 years or older. For participants without chronic kidney disease, the HR was 0.67 (95% CI, 0.51–0.89; p = 0.004), and for those with chronic kidney disease, the HR was 0.60 (95% CI, 0.49–0.73; p < 0.001). In subgroups without and with type 2 diabetes, the HRs were 0.65 (95% CI, 0.50–0.84; p = 0.001) and 0.62 (95% CI, 0.51–0.75; p < 0.001), respectively. Among participants without coronary artery disease, the HR was 0.61 (95% CI, 0.48–0.78; p < 0.001), and among those with coronary artery disease, the HR was 0.58 (95% CI, 0.48–0.71; p < 0.001). When stratified by baseline medication use, the HRs were 0.69 (95% CI, 0.44–1.07; p = 0.092) for those not receiving ACEis or ARBs and 0.63 (95% CI, 0.53–0.74; p < 0.001) for those receiving these agents. For participants not using beta-blockers, the HR was 0.55 (95% CI, 0.35–0.85; p = 0.007), and for those using beta-blockers, the HR was 0.61 (95% CI, 0.52–0.72; p < 0.001). Finally, the HRs were 0.77 (95% CI, 0.52–1.13; p = 0.184) for those not receiving MRAs and 0.54 (95% CI, 0.46–0.64; p < 0.001) for those receiving them.

### Sensitivity analysis

The association with a lower risk of MACE remained consistent when SGLT2i initiation was restricted to within 1 month (HR, 0.56; 95% CI, 0.47–0.65; P < 0.001) and within 2 months (HR, 0.57; 95% CI, 0.49–0.66; P < 0.001) after LVAD implantation (Supplementary Table S6). In component analyses of MAKE, SGLT2i use was associated with lower risks of end-stage renal disease (HR, 0.42; 95% CI, 0.29–0.60; P < 0.001), dialysis (HR, 0.22; 95% CI, 0.12–0.38; P < 0.001), and acute kidney injury (HR, 0.79; 95% CI, 0.69–0.90; P < 0.001) (Supplementary Table S7).

## Discussion

In this large, multicenter real-world cohort of patients who survived to the chronic post-LVAD phase, initiation of SGLT2i was associated with a lower risk of the primary composite endpoint between 3 months and 1 year after implantation. Notably, secondary outcomes also favored SGLT2i use, showing significant reductions in HFE, all-cause mortality, and MAKE. The direction of association was generally consistent across prespecified subgroups, although estimates were less precise among patients not receiving ACEis/ARBs or MRAs. The numerically attenuated associations among patients not receiving ACEis/ARBs or MRAs should be interpreted cautiously. Both subgroups had fewer events and wider confidence intervals, limiting statistical precision. Moreover, absence of these background therapies may identify patients with hypotension, renal dysfunction, hyperkalemia, or other treatment intolerance, potentially reflecting a clinically more vulnerable population. Accordingly, these subgroup findings may reflect limited statistical power and residual clinical differences rather than true modification of the association between SGLT2i use and outcomes.

The robustness of these findings was further supported by multiple sensitivity analyses and negative-control outcome testing, which provided some reassurance regarding unmeasured confounding, although clinically important residual confounding cannot be excluded. Importantly, there were no statistically significant differences in safety outcomes, including UTI and DKA. Although the point estimate for UTI numerically favored SGLT2i use, this finding should not be interpreted as a protective association because the confidence interval crossed unity. Moreover, the increased genitourinary infection risk associated with SGLT2is has been more consistently observed for genital mycotic infections than for bacterial UTIs (Unnikrishnan et al., 2018). Treatment-selection bias, whereby clinicians may avoid SGLT2i initiation in patients with recurrent genitourinary infections, and incomplete capture of mild infections in electronic health records may also have contributed to this observation.

Previous studies have demonstrated the prognostic benefits of continuing neurohormonal blockade after LVAD implantation. In a multicenter study of 12,144 LVAD recipients, triple neurohormonal therapy was associated with a 4-year survival rate of 56.0% compared with 43.9% among patients not receiving such therapy (HR 0.34; 95% CI 0.28–0.41), along with better quality-of-life scores and greater 6-min walk distances (McCullough et al., 2020). Similarly, in another cohort of 98 patients, ACE inhibitor or ARB use was linked to significantly lower mortality (p = 0.01) and reduced N-terminal pro-B-type natriuretic peptide levels (Vaidya et al., 2018). Together, these studies highlight the benefits of neurohormonal blockade in patients with LVAD support. More recently, SGLT2is have demonstrated consistent prognostic benefits across diverse heart-failure phenotypes, as well as in metabolic and kidney diseases, raising the question of whether these agents may also improve outcomes in the LVAD population (Piperis et al., 2024; Talha et al., 2023; Heerspink et al., 2020).

Our findings are broadly consistent with the recent analysis, which evaluate SGLT2is use among 1,312 LVAD recipients from 2014 to 2022 (Pham et al., 2025). In that study, SGLT2i therapy was associated with lower risks of all-cause mortality, all-cause hospitalization, and acute heart failure exacerbation, supporting the potential benefit of SGLT2is in this complex population. However, our study extends these observations in several important ways. First, our propensity-matched cohort included 2,636 LVAD patients, approximately twice the number included in the previous study, providing greater statistical power and event precision. Second, our analysis incorporated both subgroup and sensitivity analyses, confirming consistent benefits across key clinical characteristics such as sex, age, diabetes, chronic kidney disease, and coronary artery disease, and demonstrating robustness across multiple analytic models and negative-control outcomes. Third, unlike the prior study, which included patients from both the acute and chronic post-LVAD stages, we restricted outcome assessment to a prespecified window beginning 90 days after the index date, thereby focusing on the chronic post-LVAD phase and reducing the influence of early postoperative instability. Collectively, these methodological refinements strengthen the inference that SGLT2is use in the stable post-LVAD period is associated with improved cardiovascular and renal outcomes, including significant reductions in HFE, all-cause mortality, and MAKE.

Several mechanisms may underlie these findings. Through inhibition of the sodium–hydrogen exchanger, SGLT2is decrease intracellular sodium and calcium accumulation, thereby alleviating oxidative stress, cellular injury, and cardiomyocyte death (Uthman et al., 2018). They further improve mitochondrial function and energy efficiency while exerting anti-inflammatory and antifibrotic effects that attenuate myocardial remodeling (Packer, 2023).

In addition, SGLT2is induce osmotic diuresis and natriuresis, leading to reductions in both preload and afterload, which optimize ventricular loading conditions and overall hemodynamics (Heerspink et al., 2016; Scheen, 2020; Tang et al., 2022). These agents also promote a metabolic shift toward more energy-efficient substrates, such as free fatty acids and ketone bodies, enhancing myocardial energetic status (Packer, 2020; Verma and McMurray, 2018). Collectively, these pleiotropic effects may be particularly advantageous in patients with advanced heart failure supported by LVADs, in whom persistent neurohormonal activation, oxidative stress, and adverse loading conditions continue to drive disease progression and adverse outcomes (Chaudhry et al., 2022; Al Hazzouri et al., 2024).

Our results support the potential role of SGLT2is as part of chronic-phase pharmacologic optimization in patients with durable LVAD support. As survival after LVAD implantation continues to improve, long-term management increasingly emphasizes prevention of recurrent decompensation heart failure and preservation of renal function (Hariri et al., 2022). The observed reduction in major adverse kidney events suggests that SGLT2is may provide additional renoprotective and hemodynamic benefits, potentially mitigating right-sided congestion and improving volume stability. Although MAKE was less frequent among SGLT2i users, this association should be interpreted cautiously given the substantial residual imbalance in baseline eGFR and the inability to quantify differential laboratory-data availability between groups.

Given that most LVAD recipients are already receiving guideline-directed neurohormonal therapy, the incorporation of SGLT2is may represent the next step toward comprehensive metabolic and cardiorenal modulation in advanced heart failure. Prospective studies are warranted to confirm these associations and to clarify the optimal timing and patient selection for SGLT2is initiation.

### Limitations

This study has several limitations. First, as TriNetX is a registry-based database, the risks of patient misclassification and underrepresentation, particularly among individuals with milder disease severity or limited healthcare engagement, may affect the generalizability of our findings. In addition, reliance on diagnostic coding to identify exposures, covariates, and outcomes introduces the possibility of misclassification bias. The TriNetX propensity-score interface did not permit consolidation of overlapping hierarchical diabetes codes into a customized single binary covariate, and patient-level overlap counts were unavailable; consequently, correlated diabetes-related indicators may have contributed to residual imbalance or differential weighting within the PSM.

Furthermore, blood pressure was represented by recorded SBP because MAP and the method of blood pressure measurement were not consistently available. Because baseline covariates were assessed during the 12 months preceding the index date, some blood pressure measurements among SGLT2i users may have been obtained after LVAD implantation. In continuous-flow LVAD recipients, reduced arterial pulsatility may limit the reliability and clinical interpretation of conventional SBP measurements. Therefore, the recorded SBP values may not fully reflect clinically relevant arterial pressure in this population. To mitigate this concern, we conducted negative-control outcome analyses using unrelated clinical conditions, which demonstrated no significant group differences, suggesting minimal coding-related bias. Second, data on the duration, dosage, and adherence of SGLT2is therapy were unavailable, precluding assessment of potential dose–response relationships or the effects of long-term treatment persistence. Third, although propensity score matching was used to balance baseline characteristics, residual confounding from unmeasured variables cannot be completely excluded. To address this, we calculated E-values, which supported the robustness of our findings against potential unmeasured confounders. Fourth, the exact timing and clinical context of SGLT2i initiation relative to LVAD implantation could not be fully determined, which may have introduced heterogeneity in treatment exposure. Fifth, women were underrepresented in this study population, accounting for approximately one-quarter of the cohort. Although this distribution is consistent with prior LVAD studies, it may limit the generalizability of our findings to female patients. Further studies with more balanced sex representation are needed to confirm these results.

Sixth, detailed information on right ventricular (RV) function, including echocardiographic parameters such as tricuspid annular plane systolic excursion or RV fractional area change, was not available in the TriNetX database. Similarly, specific diagnoses of post-LVAD RV failure could not be reliably identified. Given the central role of RV dysfunction in determining prognosis after LVAD implantation, this limitation may have influenced the interpretation of our findings. Additionally, detailed information on the type and model of LVAD devices (e.g., HeartMate 3, HeartWare, or other less commonly used devices) was not available in the TriNetX database. Given that newer-generation devices, particularly HeartMate 3, have demonstrated improved clinical outcomes compared with earlier devices, residual confounding related to device heterogeneity cannot be excluded. Although the study period spans years during which newer devices became predominant, this limitation should be considered when interpreting the findings. Additionally, restricting SGLT2i initiation to within 1 and 2 months after LVAD implantation reduced the temporal separation between implantation and treatment initiation, these sensitivity analyses did not fully eliminate potential immortal time bias arising from the use of group-specific index dates.

Seventh, substantial residual imbalance in baseline kidney function remained after propensity score matching, with a higher mean eGFR in the SGLT2i group. Because kidney function is strongly associated with subsequent cardiovascular and renal outcomes, this imbalance may have favored the SGLT2i group and may partly explain the observed reductions in MAKE and other clinical outcomes. Although the proportion of patients with eGFR <15 mL/min/1.73 m^2^ was well balanced, residual confounding across the broader spectrum of kidney function cannot be excluded. In addition, variable-specific missingness for laboratory and vital-sign measurements could not be quantified within the matched cohorts using the available aggregate TriNetX analytical environment. Because the two exposure groups were indexed at different clinical time points, differential availability and timing of laboratory testing, particularly serum creatinine measurements used to derive eGFR, may have contributed to the observed residual imbalance in kidney function. This potential ascertainment bias could not be fully assessed and should be considered when interpreting the renal findings.

Finally, cause-specific mortality data were not available, preventing differentiation between cardiovascular and non-cardiovascular deaths and potentially leading to endpoint misclassification.

## Conclusion

In conclusion, among patients with advanced heart failure who survived to the chronic post-LVAD phase, treatment with SGLT2is was associated with a significantly lower incidence of the primary composite endpoint, HFE, all-cause mortality, and MAKE between 3 months and 1 year after implantation. These findings provide real-world evidence supporting the potential chronic-phase benefits of SGLT2is therapy in this high-risk population. Future prospective studies are warranted to validate these associations, elucidate underlying mechanisms, and determine the optimal timing and patient selection for initiating SGLT2is following LVAD implantation.

## Data Availability

The dataset is subject to restrictions under TriNetX data-sharing policies. The investigators had access only to aggregated and de-identified data and did not have access to individual-level patient data or protected health information. Therefore, the underlying patient-level data cannot be publicly shared by the authors. Access to the data requires approval through the TriNetX platform and compliance with applicable privacy, institutional, and regulatory requirements. Requests to access the dataset can be made through the TriNetX platform. Further information is available at the TriNetX website: https://trinetx.com. Investigators may also contact TriNetX support at support@trinetx.com for information regarding data access procedures.
