# Supplementary material for: SGLT2 inhibitors and outcomes in the chronic Post-LVAD phase: a multicenter real-world cohort study
Source: Front Pharmacol. 2026 Sep 8;17:1922067. doi: 10.3389/fphar.2026.1922067 (PMC13593425; doi:10.3389/fphar.2026.1922067)
Supplement: Supplementary file 1 [file Supplementaryfile1.docx]

**Table S1.** Demographic, diagnostic, procedural, medication, visit, and laboratory codes utilized in the definition of the cohorts.

| **Category** | **Code** | **Description** |
| --- | --- | --- |
| **Index drug** | | |
| medication | NLM:RXNORM:1488564 | dapagliflozin |
| medication | NLM:RXNORM:1545653 | empagliflozin |
| **LVAD** | | |
| procedure | UMLS:CPT:33975 | Insertion of ventricular assist device; extracorporeal, single ventricle |
| procedure | UMLS:ICD10PCS:02HA3QZ | Insertion of Implantable Heart Assist System into Heart, Percutaneous Approach |
| procedure | UMLS:CPT:33979 | Insertion of ventricular assist device, implantable intracorporeal, single ventricle |
| procedure | UMLS:ICD10PCS:02HA0QZ | Insertion of Implantable Heart Assist System into Heart, Open Approach |
| procedure | UMLS:ICD10PCS:02HA4QZ | Insertion of Implantable Heart Assist System into Heart, Percutaneous Endoscopic Approach |

**Table S2.** Covariates included in the propensity score model and their coding and functional forms.

| **Code** | **Description** | **Functional form** |
| --- | --- | --- |
| AI | Age at Index | Continuous |
| 2106-3 | White | Binary indicator |
| UNK | Unknown Race | Binary indicator |
| F | Female | Binary indicator |
| 2054-5 | Black or African American | Binary indicator |
| M | Male | Binary indicator |
| N18 | Chronic kidney disease (CKD) | Binary indicator |
| E11.2 | Type 2 diabetes mellitus with kidney complications | Binary indicator |
| E11.3 | Type 2 diabetes mellitus with ophthalmic complications | Binary indicator |
| E11.4 | Type 2 diabetes mellitus with neurological complications | Binary indicator |
| E11.5 | Type 2 diabetes mellitus with circulatory complications | Binary indicator |
| E11 | Type 2 diabetes mellitus | Binary indicator |
| F17 | Nicotine dependence | Binary indicator |
| I48 | Atrial fibrillation and flutter | Binary indicator |
| J44.9 | Chronic obstructive pulmonary disease, unspecified | Binary indicator |
| K74 | Fibrosis and cirrhosis of liver | Binary indicator |
| C07 | BETA BLOCKING AGENTS | Binary indicator |
| C03D | ALDOSTERONE ANTAGONISTS AND OTHER POTASSIUM-SPARING AGENTS | Binary indicator |
| A10BJ | Glucagon-like peptide-1 (GLP-1) analogues | Binary indicator |
| BL117 | PLATELET AGGREGATION INHIBITORS | Binary indicator |
| CV800 | ACE INHIBITORS | Binary indicator |
| CV805 | ANGIOTENSIN II INHIBITOR | Binary indicator |
| 9037 | Hemoglobin A1c/Hemoglobin.total in Blood | Continuous + binary threshold indicator |
| 8001 | Glomerular filtration rate/1.73 sq M.predicted [Volume Rate/Area] in Serum, Plasma or Blood by Creatinine-based formula (MDRD) | Continuous + binary threshold indicator |
| 9002 | Cholesterol in LDL [Mass/volume] in Serum or Plasma | Continuous + binary threshold indicator |
| 9085 | Blood Pressure, Systolic | Continuous + binary threshold indicator |

E11 and the complication-specific E11.2–E11.5 categories are hierarchical diagnostic codes and may overlap within individual patients; these categories should therefore not be interpreted as mutually exclusive.

**Table S3.** Definitions of outcomes coding used in this study.

| **Code** | **Description** |
| --- | --- |
| **MACE** | |
| UMLS:ICD10CM:R99 | Ill-defined and unknown cause of mortality |
| Deceased | Deceased |
| NLM:VA:CV701 | THIAZIDES/RELATED DIURETICS (Route: Injectable Product) |
| NLM:VA:CV702 | LOOP DIURETICS (Route: Injectable Product) |
| UMLS:ICD10CM:J81 | Pulmonary edema |
| **HFE** | |
| NLM:VA:CV701 | THIAZIDES/RELATED DIURETICS (Route: Injectable Product) |
| NLM:VA:CV702 | LOOP DIURETICS (Route: Injectable Product) |
| UMLS:ICD10CM:J81 | Pulmonary edema |
| **All-cause mortality** | |
| UMLS:ICD10CM:R99 | Ill-defined and unknown cause of mortality |
| Deceased | Deceased |
| **MAKE** | |
| UMLS:ICD10CM:N18.6 | End stage renal disease |
| UMLS:CPT:1006747 | Hemodialysis Access, Intervascular Cannulation for Extracorporeal Circulation, or Shunt Insertion Procedures on Arteries and Veins |
| UMLS:CPT:1012752 | Hemodialysis Procedures |
| UMLS:ICD10CM:N17 | Acute kidney failure |
| UMLS:ICD10CM:N18.5 | Chronic kidney disease, stage 5 |
| **DKA** | |
| UMLS:ICD10CM:E11.1 | Type 2 diabetes mellitus with ketoacidosis |
| UMLS:ICD10CM:E13.1 | Other specified diabetes mellitus with Ketoacidosis |
| **UTI** | |
| UMLS:ICD10CM:N39.0 | Urinary tract infection, site not specified |
| **Hernia** | |
| UMLS:ICD10CM:K40-K46 | Hernia |

**Table S4**. Negative and safety outcomes control.

| Outcome | HR (95% CI) | *P* value |
| --- | --- | --- |
|  |  |  |
| Safety outcome |  |  |
| UTI | 0.69 (0.45,1.06) | 0.091 |
| DKA | 1.25 (0.71,2.23) | 0.437 |
| Negative outcome |  |  |
| Hernia | 0.96 (0.73,1.26) | 0.748 |

**Table S5.** Era-restricted sensitivity analysis.

| Outcome | HR (95% CI) | *P* value |
| --- | --- | --- |
|  |  |  |
| 2014-2019 | 0.60 (0.50,0.72) | <.001 |
| 2020-2025 | 0.62 (0.54,0.72) | <.001 |

**Table S6.** Sensitivity analyses restricting SGLT2i initiation to within 1 and 2 months after LVAD implantation.

| SGLT2i initiation after LVAD implantation | HR (95% CI) | *P* value |
| --- | --- | --- |
|  |  |  |
| Within 1-month | 0.56 (0.47,0.65) | <.001 |
| Within 2-months | 0.57 (0.49,0.66) | <.001 |

**Table S7.** Components of major adverse kidney events.

| Outcome | HR (95% CI) | *P* value |
| --- | --- | --- |
|  |  |  |
| End-stage renal disease | 0.42 (0.29,0.60) | <.001 |
| Dialysis | 0.22 (0.12,0.38) | <.001 |
| Chronic kidney disease stage 5 | Not estimable | - |
| Acute kidney injury | 0.79 (0.69,0.90) | <.001 |

Not estimable: Not reported owing to TriNetX suppression of small cell counts
